# Efficacy of indocyanine green fluorescence-based near-infrared angiography in assessing intraoperative wound perfusion for bone and soft-tissue surgery

**DOI:** 10.1302/2633-1462.67.BJO-2024-0248.R1

**Published:** 2025-07-11

**Authors:** Han Wang, Xiaodong Tang, Tao Ji, Dasen Li, Huayi Qu, Zhiye Du, Wei Guo

**Affiliations:** 1 Musculoskeletal Tumor Center, Peking University People’s Hospital, Beijing, China

**Keywords:** Indocyanine green, Wound perfusion, Angiography, Bone and soft-tissue tumour, soft-tissue surgery, angiography, wounds, soft-tissue tumour, fluorescence angiography, clinical outcomes, reoperations, blood, Multivariate analysis, wound healing

## Abstract

**Aims:**

Wound complication is common in bone and soft-tissue tumour surgery. Proper wound healing requires robust blood perfusion. However, intraoperative assessment of perfusion is difficult, and lacks methods with good accuracy. This study aimed to explore the efficacy of indocyanine green fluorescence angiography (ICGA) in intraoperatively assessing wound perfusion and predicting postoperative wound necrosis and clinical outcomes.

**Methods:**

A total of 22 patients with orthopaedic oncological diseases were enrolled in this study from August 2021 to December 2022. All patients were deemed to have high risk of postoperative necrosis but normal wound appearance during surgery. ICGA was performed intraoperatively to assess the perfusion status of the wound. A novel system, called the Fluorescence Perfusion Scale (FPS), was proposed, consisting of three types of fluorescence angiography features corresponding to different perfusion statuses. Patient- and provider-related risk factors were analyzed. The relationship between clinical outcomes and FPS types was analyzed.

**Results:**

Wound necrosis occurred in ten of 22 patients (46%). According to the FPS, seven patients (32%) showed type 1, seven patients (32%) showed type 2, and eight patients (36%) showed type 3 angiography, respectively. The uni- and multivariate analysis indicated that FPS type 1 or 2 is the only independent risk factor for wound necrosis. The necrosis rate showed a significant difference between FPS types (p＜0.001). The rate of reoperation of patients of type 1 was significantly higher than that of type 2 (85% compared 0%, p = 0.005).

**Conclusion:**

Wound perfusion in bone and soft-tissue surgery can be assessed by ICGA and graded by the FPS system, which can predict postoperative necrosis and clinical outcomes.

Cite this article: *Bone Jt Open* 2025;6(7):796–806.

## Introduction

Wound complications are not uncommon in bone and soft-tissue tumour surgeries due to the necessary wide dissection or planned resection of subcutaneous tissue/skin. These complications may cause surgical site infection (SSI), delayed adjuvant therapy, and reoperations.^1,2^ Risk factors for wound complications include patient-related factors (such as diabetes, smoking, and vascular disease) and provider-related factors (such as long operating time and improper skin incision/suture).^1^ All these factors are based on the concept that robust perfusion is essential to proper wound healing.^3^ However, intraoperative assessment of wound perfusion is challenging, and currently relies on the evaluation of clinical examination such as wound colour, temperature, capillary reperfusion, and bleeding, which can be inaccurate.^4,5^

Near-infrared (NIR) fluorescence angiography with indocyanine green (ICG) allows real-time visualization of skin blood perfusion with a large scanning range in the operating theatre.^6,7^ Although other methods have been applied to assess tissue perfusion, such as colour-duplex ultrasound and intraoperative blood gas analysis, ICG angiography (ICGA) provides a direct observation of the wound and is gaining popularity.^5,8,9^ It has been widely used in the assessment of perfusion and perforator mapping of flap surgery.^10^ However, the role of ICGA in evaluating wound perfusion during bone and soft-tissue surgery remains unclear.

Another problem yet to be solved is how to analyze the ICGA images.^11^ Analysis methods include non-quantitative and quantitative analysis, each with its own advantages and disadvantages. Thus, based on the characteristics of bone and soft-tissue tumour surgeries, we proposed a novel grading system called the Fluorescence Perfusion Scale (FPS), which is semiquantitative. This study aimed to explore whether wound perfusion can be assessed by ICGA and graded by FPS in bone and soft-tissue surgery; and whether FPS grading was associated with postoperative wound necrosis and clinical outcomes.

## Methods

### Study design and ethics

A retrospective single-institute study was conducted in the accordance with the Declaration of Helsinki^12^ and was approved by the Ethics Committee in our institute. All patients provided informed consent.

### Patients and data collection

A total of 22 patients with musculoskeletal oncology diseases who underwent surgery from August 2021 to December 2022 in our centre were enrolled in this study. This series included 15 males (68%) and seven females (32%) with a mean age of 39 years (11 to 73). The pathology consisted of 18 cases with malignant diseases (81%) and three cases with benign diseases (19%). The median follow-up was 70 days (IQR 21 days to one year). General information is shown in Table I. All patients were confirmed to have no history of iodine allergy.

**Table I.** General information of enrolled patients.

Patient No.	Sex	Age, yrs	BMI, kg/m^2^	Pathology	Tumour volume, cm^3^	Operating time, mins	Preoperative embolization of tumour artery	Performed surgery	Surgical site	Implants	Neoadjuvant therapy	Adjuvant therapy	Postoperative medication	Suture	FPS type	Postoperative necrosis	Clinical outcome
1	M	14	29	Osteosarcoma	140.0	275	N	Tumour resection	Calcaneus	Y	C	I	D	St + VM + SI	1	Y	Unhealed with twice debridement and finally amputation
2	M	71	26	Chordoma	1,693.0	335	N	Tumour resection	Sacrum	Y	R	N	N	St + VM + SI	1	Y	Healed with once debridement and removing internal fixation
3	F	20	18	Osteosarcoma	422.4	575	Y	Tumour resection	Pelvis	Y	C	C + I	D	St	1	Y	Healed with once debridement
4	M	43	26	Hemangiosarcoma	967.6	500	N	Tumour resection	Pelvis	Y	C	C	D	St + VM + SI	1	Y	Healed with once debridement
5	F	25	20	Fibrosarcoma	1,142.8	380	Y	Tumour resection	Sacrum	Y	N	N	N	St + VM + SI	1	Y	Healed with twice debridement and once internal fixation revision
6	M	53	20	Epithelioid sarcoma	12.3	235	N	Tumour resection	Hand	N	C	C + T	D + V	VM + SI	1	Y	Live with stable necrosis lesion with long-term wound dressing
7	M	37	24	Chondrosarcoma	507.7	310	N	Amputation	Front and middle foot	N	N	N	N	St + VM + SI	1	Y	Healed with once debridement
8	M	15	16	Osteosarcoma	20.0	182	N	Tumour resection	Calcaneus	Y	C	C	D	VM + SI	2A	Y	Healed with long-term wound dressing
9	F	35	19	Rhabdomyosarcoma	8.8	302	N	Tumour resection	Ankle	Y	C	C	D	VM + SI	2A	Y	Healed with long-term wound dressing
10	M	56	20	MPNST	3,473.8	160	N	Tumour resection	Gluteus	N	N	N	N	VM + SI	2A	Y	Healed with long-term wound dressing
11	M	55	26	Undifferentiated sarcoma	838.0	180	N	Tumour resection	Pelvis	N	C	C	D	VM + SI	2B	N	Healed with no additional intervention
12	F	55	23	Giant cell tumour of bone	572.2	205	N	Debridement due to endoprothesis infection	Tibia	Y	N	N	D	VM + SI	2B	N	Healed with postoperative vasodilators and long-term wound dressing
13	F	14	20	Osteosarcoma	4.3	55	N	Tumour resection	Chest wall	N	T	T	N	VM + SI	2B	N	Healed with no additional intervention
14	F	11	16	Osteosarcoma	229.6	35	N	Debridement due to infection	Tibia	N	N	N	N	VM + SI	2B	N	Healed with removing the overtight stitches and postoperative vasodilators
15	M	53	22	Undifferentiated sarcoma	149.9	500	Y	Tumour resection	Sacrum	Y	C + R	C	N	VM + SI	3	N	Healed with no additional intervention
16	F	62	23	Bone infarction	0.5	135	N	Tumour resection	Cuneiform bone	Y	N	N	N	St	3	N	Healed with no additional intervention
17	M	11	19	Osteosarcoma	9.5	30	N	Tumour resection	Chest wall	N	C	C	N	St	3	N	Healed with no additional intervention
18	M	73	23	Bone metastasis	16.2	320	N	Tumour resection	Femur	Y	T	T	N	Sc	3	N	Healed with no additional intervention
19	M	34	28	Eosinophilic granuloma	13.9	155	N	Tumour resection	Sacrum	N	N	N	N	Sc	3	N	Healed with no additional intervention
20	M	60	23	Chordoma	2,418.8	205	N	Tumour resection	Sacrum	N	T	N	N	St + VM + SI	3	N	Healed with no additional intervention
21	M	20	19	Giant cell tumour of bone	114.5	240	N	Tumour resection	Sacrum	Y	N	N	N	St + VM + SI	3	N	Healed with no additional intervention
22	M	58	23	Ewing’s sarcoma	106.1	207	N	Tumour resection	Neck	N	C	C	N	SI	3	N	Healed with no additional intervention

C, chemotherapy; D, decongestant; FPS, fluorescence perfusion scale; I, immunotherapy; MPNST, malignant peripheral nerve sheath tumour; N, no; R, radiotherapy; Sc, subcuticular running suture; SI, simple interrupted suture; St, staples; T, targeted therapy; V, vasodilator; VM, vertical mattress suture; Y, yes.

Patient- and provider-related risk factors for necrosis were obtained from electronic medical records. Patient-related factors included BMI, tumour volume, location, history of heart disease, vascular disease, diabetes, and steroid drug use. Provider-related factors included surgery duration, artery reconstruction status, preoperative embolization status, neoadjuvant and adjuvant therapy, and suture method. Tumour volume was calculated using the formula: height × width × length × 0.52. Tumour height, width, and length were obtained from preoperative CT or MRI.

### Wound closure, management, and follow-up

The wounds were closed to restore normal anatomy as much as possible based on tissue defect. The suture method was chosen based on the wound tension, soft-tissue status, and surgeon preference. Generally, for sites with great tension, simple interrupted or vertical mattress suture with silk was preferred; for sites with lower tension, staples or subcuticular running suture was preferred. Wound dressing was performed with sterile gauze according to clinical routine every three days postoperatively until all sutures were removed and all scabs had fallen off. Vasodilators were used according to surgeon preference, healing status, and bleeding risk. Whether to perform neoadjuvant or adjuvant therapy was decided based on tumour pathology, tumour size, treatment guideline, anatomy, and surgical margin status. Artery reconstruction was performed based on the artery status and surgeon’s decision. All patients were followed to monitor wound healing with the following endpoints: 1) wound completely healed; or 2) one year after surgery.

### ICG preparation

ICG (Ruida Pharmaceutical, China) was prepared by dissolving 25 mg of powder in 10 ml sterile water. The powder was confirmed to be completely dissolved. The dye was always kept in the dark and was administered within ten minutes after preparation.

### Surgical procedure and fluorescence angiography

The surgery was conducted according to clinical routine based on the disease and therapeutic plan. ICGA was performed after all sutures were completed via intravenous injection of all 10 ml of prepared ICG solution through a peripheral vein followed by 10 ml flush with normal saline. All lights in the operating theatre were shut down, and vital signs were monitored closely during the entire ICGA process. NIR fluorescence imaging was carried out with H3800 system (Digital Precision Medicine, China), which allows NIR imaging using a hand-held camera. The distance between the camera and wound was kept at 20 to 30 cm. It usually took 10 to 20 seconds for ICG to reach the surgical site, and 15 to 30 seconds to show maximal fluorescence. The entire angiography process was recorded by the equipment in black and white mode. Representative images was captured and analyzed when the fluorescence was strongest. Coloured merging images were not analyzed, in order to avoid the influence of merging the algorithm during image processing.

### Fluorescence perfusion scale

**Fig. 1:**
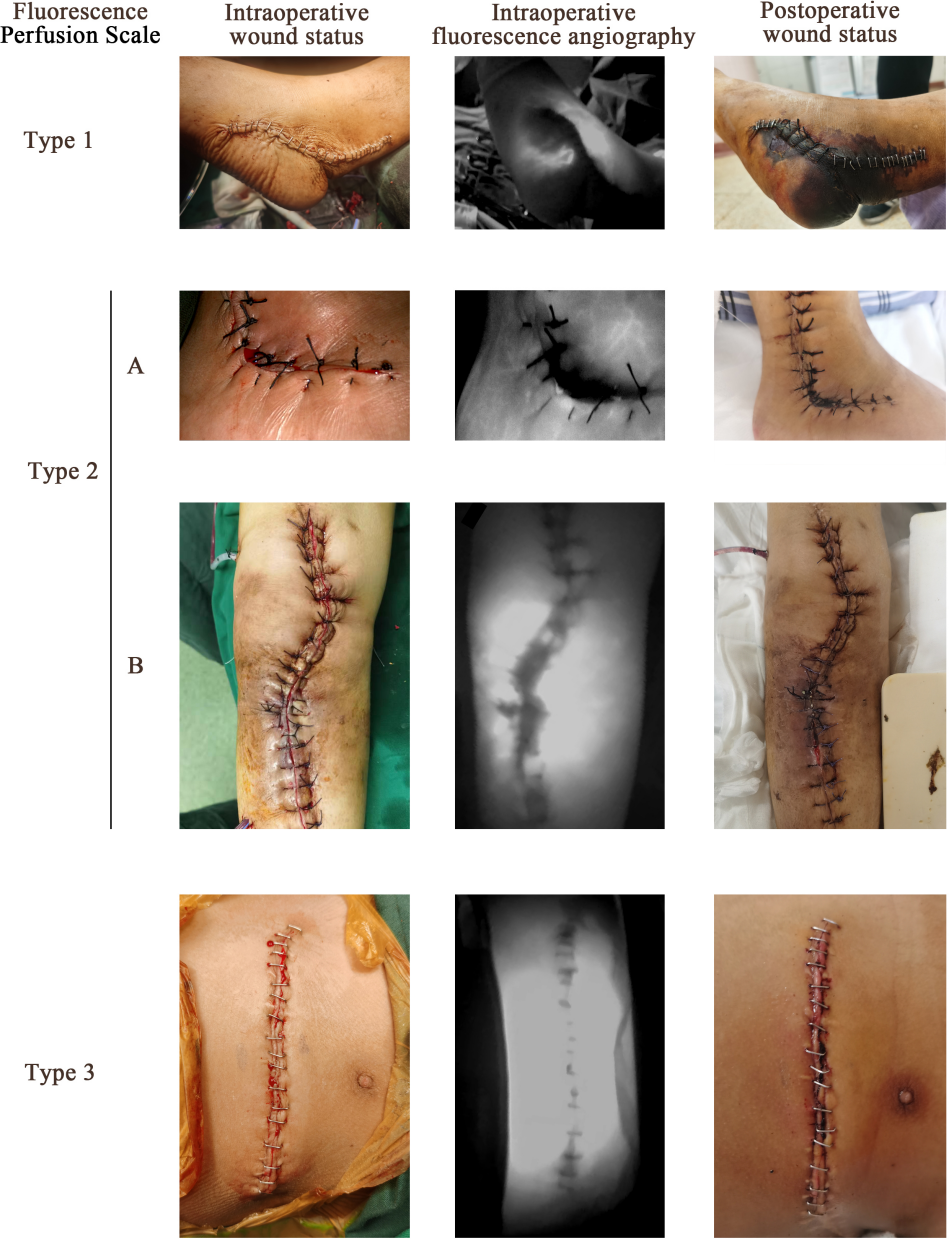
Representative imaging of intraoperative wound status (left column), indocyanine green (ICG) angiography (middle column), and postoperative wound status (right column) of different Fluorescence Perfusion Scale (FPS) types. The type 1 imaging was from patient No. 1. This patient showed a normal appearance of intraoperative wound but a hypofluorescent area beyond the stitches line in ICG angiography. Postoperative necrosis occurred. The type 2A imaging was from patient No. 9. This patient also showed a normal wound appearance but with an asymmetric hypofluorescent area within the stitches line. A small area of postoperative necrosis occurred. The type 2B imaging was from patient No. 14. This patient showed normal wound appearance but with a symmetric hypofluorescent area within the stitches line. No postoperative necrosis occurred. The type 3 imaging was from patient No. 17. This patient showed normal appearance of wound and no hypofluorescent area. No postoperative necrosis occurred.

The FPS system was proposed based on the range of hypofluorescent areas in ICGA, and consists of three types corresponding to different perfusion status (Table II and Figure 1). Symmetric hypofluorescent areas confined to the stitch lines were thought to be caused by overtight sutures. Hypofluorescent areas beyond the stitch lines, or asymmetrically distributed on both sides of the wound, were related to non-sutural causes, such as extensive damage to subcutaneous tissue/skin or embolization of supply vessels. The non-sutural causes are common in the surgery of bone and soft-tissue tumour, but are hard to assess the degree of severity intraoperatively.

**Table II.** The Fluorescence Perfusion Scale (FPS) system.

FPS	Fluorescence feature	Perfusion status	Necrosis and clinical outcome prediction
Type 1	Hypofluorescent area beyond the stitches line.	Seriously disrupted perfusion due to non-sutural-related reason with or without overtight suture.	Large range of necrosis will be very likely to occur in hypofluorescent areas which usually needs debridement or flap transferring/grafting.
Type 2	Hypofluorescent area confined to the stitches line. This type can be further classified into two subtypes: 2A: Hypofluorescent area shows obvious asymmetry 2B: Hypofluorescent area symmetrically distributed in both sides of wound.	Slightly disrupted perfusion due to overtight suture. For the side with darker fluorescence of type 2A, non-sutural related ischaemia may coexist.	Necrosis may occur, especially in the darker side of type 2A cases. However, the range of necrosis may be relatively small and usually able to heal with prolonged wound dressing and careful follow-up.
Type 3	No hypofluorescent area.	Normal perfusion.	Necrosis will not occur.

### Statistical analysis

Univariate analysis was performed first. For continuous variables, independent-samples *t*-test was used to detect the differences between groups. Mann-Whitney U test was used for continuous variables that were not normally distributed. For all categorical variables, the chi-squared test was adopted. Variables with p-values less than 0.10 were selected for multivariate logistic analysis. During multivariate logistic regression, a statistical problem called ‘perfect separation’ occurred, as FPS type 3 always predicted no postoperative necrosis.^13^ Thus, a virtual patient was introduced as noise data to break the separation with a tendency to make the results non-significant.^13^ This patient underwent necrosis with benign disease, with an operating time less than 144 minutes and FPS type 3. A significant result in this case indicates significance in the original data. SPSS v. 19.0 (IBM, USA) was used for statistical analysis. The significance level was set at p < 0.05. All p-values were obtained from two-sided tests.

## Results

### Wound necrosis and analysis of related risk factors

Wound necrosis occurred in 10/22 patients (46%). All patients were deemed to have normal wound perfusion via intraoperative clinical examination. Univariate analysis indicated that three risk factors (surgery duration, tumour pathology, and FPS type) showed p-values < 0.100 and were included in multivariate analysis (Table III). The multivariate logistic analysis indicated that FPS type 1 or 2 is an independent risk factor for wound necrosis (Table IV).

**Table III.** Univariate analysis of necrosis risk.

Factor	Necrosis		p-value
	Yes (n = 10)	No (n = 12)	
Mean age, yrs (SD)	37 (19)	42 (23)	0.567
Mean BMI, kg/m^2^ (SD)	22 (4)	22 (3)	0.790
Median tumour volume, cm^3^ (IQR)	465 (18 to 1,280)	110 (10 to 486)	0.187
**Heart disease, % (n)**			
No	43 (9)	57 (12)	0.455
Yes	100 (1)	0 (0)	
**Smoker, % (n)**			
No	48 (10)	52 (11)	> 0.999
Yes	0 (0)	100 (1)	
**Vascular disease, % (n)**			
No	46 (10)	55 (12)	N/A
**Steroid use, % (n)**			
No	43 (9)	57 (12)	0.455
Yes	100 (1)	0 (0)	
**Diabetes, % (n)**			
No	43 (9)	57 (12)	0.455
Yes	100 (1)	0 (0)	
**Location, % (n)**			
Trunk	38 (5)	62 (8)	0.666
Limb	56 (5)	45 (4)	
**Pathology, % (n)**			
Malignant	56 (10)	44 (8)	0.096
Benign	0 (0)	100 (4)	
Mean operating time, mins (SD)	325 (131)	179 (125)	0.015
Operating time ≤ 144 mins, % (n)	18 (2)	82 (9)	0.010
Operating time＞144 mins, % (n)	73 (8)	27 (3)	
**Artery reconstruction, % (n)**			
No	43 (9)	57 (12)	0.455
Yes	100 (1)	0 (0)	
**Preoperative embolization, % (n)**			
No	42 (8)	58 (11)	0.571
Yes	67 (2)	33 (1)	
**Neoadjuvant therapy, % (n)**			
None	38 (3)	63 (5)	0.409
**Radiotherapy, % (n)**	100 (1)	0 (0)	
Other	46 (6)	54 (7)	
**Adjuvant therapy, % (n)**			
None	40 (4)	60 (6)	0.691
Yes	50 (6)	50 (6)	
**Suture, % (n)**			
St + VM + SI	71 (5)	29 (2)	0.332
St	33 (1)	67 (2)	
VM + SI	44 (3)	56 (5)	
Sc	0 (0)	100 (2)	
SI	0	100 (1)	
**FPS, % (n)**			
1/2	71 (10)	29 (4)	0.002
3	100 (8)	0(0)	

N/A, not applicable; Sc, subcuticular running suture; SI, simple interrupted suture; St, staples; VM, vertical mattress suture.

**Table IV.** Multivariate analysis of necrosis risk.

Factor	p-value	OR	95% CI
Operating time > 144 mins	0.062	0.090	0.070 to 1.129
Malignancy	0.789	0.652	0.028 to 15.059
FPS type 1 or 2	0.030	29.181	1.378 to 617.723

FPS, Fluorescence Perfusion Scale; OR, odds ratio.

### FPS system and corresponding necrosis and clinical outcomes

The ICGA features of patients were classified according to the FPS system. Seven patients (32%) showed type 1 angiography, while seven (32%) and eight (36%) showed type 2 and 3 angiography, respectively.

All seven cases with FPS type 1 angiography had postoperative skin necrosis, six (85%) of which underwent debridement at least once. One patient (No. 1) refused the advice of flap transplantation due to the large necrosis range that could not be handled by simple debridement, and eventually underwent amputation. Although necrosis occurred, one patient (No. 6) refused advice of debridement due to the relatively small (about 2 × 1 cm) and tolerable necrosis range. At the last follow-up, the patient was living with a stable and dry scab with a tendency to spontaneously fall off.

All patients with FPS type 2 had healed wounds without reoperation. However, three patients (No. 8, 9, and 10) classified as type 2A angiography developed small areas of wound necrosis. Surgeons decided on conservative treatments (prolonged wound dressing), and all three patients had a healed wound within three months post-surgery. Other patients were classified as FGS type 2B and had no postoperative necrosis. One patient (No. 12) was given vasodilators and prolonged wound dressing due to high wound risk with concurrent infection. For another patient (No. 14), two overtight sutures were removed intraoperatively under ICGA guidance, after which the hypofluorescent area shrank, and resuturing with less tension was performed. This patient had normal postoperative healing. The wounds of the other two patients (No. 11 and 13) healed normally. All eight patients with FGS type 3 had normally healed wounds.

**Fig. 2:**
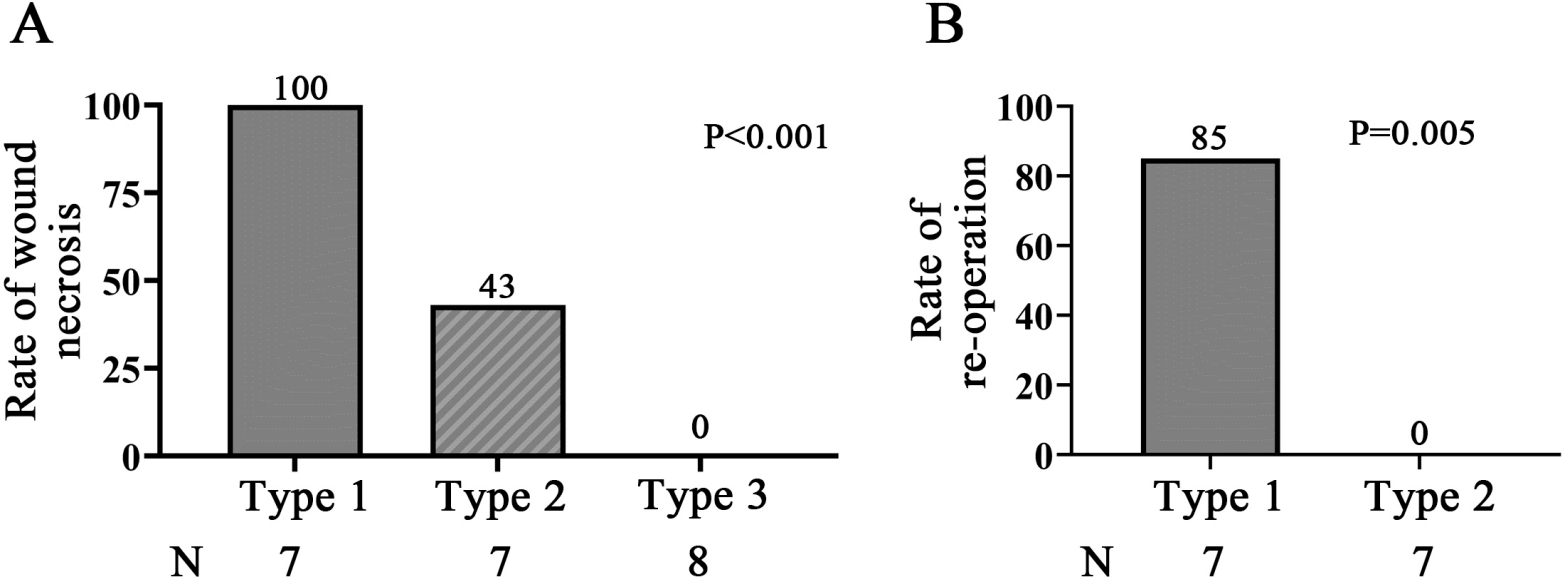
The rate of necrosis and reoperation showed a significant difference between Fluorescence Perfusion Scale (FPS) types. a) The necrosis rate showed a significant difference between FPS types. b) The reoperation rate of FPS type 1 was significantly higher than that of FPS type 2.

The necrosis rate showed a significant difference between FGS types (Figure 2a) (p < 0.001, chi-squared test). The reoperation rate showed a significant difference between FGS type 1 and type 2 (Figure 2b) (85% compared with 0%, p = 0.005, chi-squared test). The prediction of necrosis and clinical outcome is summarized in Table II.

## Discussion

Wound necrosis is a troubling complication in musculoskeletal tumour surgery, potentially leading to infection and delayed adjuvant therapy.^1,2^ Adequate perfusion is essential for a normal healing process. Among the innovative technologies currently used for intraoperative perfusion assessment, from noninvasive to minimally invasive and from infrared to ultrasound, ICG-based fluorescence angiography is the most used method with a good safety record.^4^ However, its role in wound perfusion assessment in orthopaedic oncology remains unclear. In this study, we proposed an ICGA scaling system called FPS to assess wound perfusion status and explored its role in wound necrosis and outcome prediction through a retrospective study of 22 patients with bone and soft-tissue tumour.

For ethical considerations to avoid unnecessary skin resection, no intraoperative measures were taken in this series, except for the removal and resuturing of overtight stitches under the guidance of fluorescence in one patient (No. 14). Therefore, the results well reflect the unbiased natural outcomes of different ICGA types. Based on these results, intraoperative proposals are summarized in Table V to guide the future application of this technique.

**Table V.** Intraoperative proposals according to the Fluorescence Perfusion Scale (FPS).

FPS	Proposals
Type 1	Carefully analyze the cause of ischaemia: If suspect tension ischaemia, remove the possible responsible suture and see if hypofluorescent areas completely disappear. If suspect too wide subcutaneous tissue dissection, resect hypofluorescent areas. When these areas are too large to remove immediately, wait 7 to 10 days to sharpen the margin of necrosis and plan proper second operation.
Type 2	Remove the possible responsible suture and see if hypofluorescent areas completely disappear. If not: for 2A cases, resect hypofluorescent areas in the darker side. When these areas are not suitable to be removed immediately, make sure the patient can tolerate long-term surveillance and/or delayed adjuvant therapy because he or she may need prolonged wound dressing. for 2B cases, repercussive drugs and/or vasodilators may be considered postoperatively.
Type 3	No need to take measures.

Among reported methods to evaluate wound perfusion, ICGA is easy to use, fast, and reproducible, and becoming increasingly popular.^4,14,15^ It provides direct observation of the tissue perfusion status with a thickness of 3 to 5 mm.^7^ Although patient- and surgery-related risk factors for wound necrosis have been reported, our results showed that FPS type is the only independent risk factor. The reason for this is that those risk factors do not directly visualize wound perfusion status. Compared with indirect indicators, ICGA directly visualizes wound perfusion status and thus has better reliability. It has been reported that high-risk patients, such as smokers, obese individuals, and those with vascular diseases, can benefit from ICGA.^5^ Other methods are not as popular as ICGA:^4^ Fluorescein angiography has poor repeatability due to a long half-life of 60 minutes;^16^colour Doppler ultrasound exhibits operator dependence and can easily have false positivity;^17^ and intraoperative blood gas analysis requires multiple complex blood collections.^4,18^

ICGA imaging analysis methods are still controversial. Earlier studies were mainly based on the surgeon’s subjective interpretation of ICGA.^19,20^ One study used software to quantitatively analyze the fluorescence intensity (FI) of ICGA to increase perfusion evaluation accuracy.^21^ However, the surgeon’s subjective interpretation may bring observational bias, and FI analysis can be influenced by imaging conditions and choice of region of interest (ROI). Thus, results in the literature vary and lack external applicability.^11,22^ The FPS system proposed in this study does not rely on subjective judgement or unstable FI analysis. It simplifies the analysis process and provides an easy-to-use reference standard of perfusion assessment with ICGA.

In plastic and reconstructive surgery, ICGA has been widely used in assessment of free flap perfusion, anastomotic patency, and perforator mapping.^10^ However, orthopaedic surgery differs significantly from plastic surgery in terms of disease, surgical approach, and principles. Some studies have reported good results from using this technique in tissue perfusion assessment in acute paediatric trauma,^15^ complex knee reconstruction,^23^ and upper limb surgeries.^24^ Our results also showed good value of ICGA in perfusion assessment in orthopaedic oncology, consistent with those reports. However, the literature mostly relies on the surgeon’s subjective interpretation and lacks a summarized ICGA feature. The FPS system proposed in this study could help to standardize ICGA feature descriptions in future reports, and may be applied to fields beyond orthopaedic oncology. For example, cases 1 and 3 in Ghareeb et al’s^24^ report can both be classified as FPS type 1 angiography, both of which developed necrosis. Case 7 in Wyles et al’s^23^ report initially showed FPS type 1 angiography, but turned to FPS type 3 after releasing overtight sutures, and this patient eventually healed normally.

Consistent with previous literature, our results indicated that ICGA tends to underestimate wound perfusion due to its hypersensitivity to ischaemia.^25^ Thus, the absence of hypofluorescent areas indicates robust perfusion status (shown in type 3 in Figure 1). However, slight hypoperfusion not necessarily causing necrosis may result in an obvious hypofluorescent area (as shown in the type 2B case in Figure 1). Similar findings have been reported in other studies, where reduced fluorescent perfusion was observed within the anastomotic staple line in virtually all cases of intestinal anastomosis but resulted in no necrosis.^25,26^ The eventual necrotic area was usually smaller than the hypofluorescent area (shown in the type 1 and 2A cases in Figure 1). Therefore, radical resection of all hypofluorescent area theoretically ensures complete normal perfusion of the wound. However, it should be noted that no tension ischaemia should be generated after skin resection. We suggest careful consideration and analysis of the reason for the ischaemia before resection of hypofluorescent areas (as suggested in Table V). When this area is too large to completely resect immediately, a compromised resection of a smaller area with weak fluorescence is also acceptable (mainly used in FGS type 2A).

There are some limitations to this work. First, the relatively small sample size prevented subgroup analysis for different types of surgery and surgical sites, which may show different ICGA features and influence the accuracy of FPS. Additionally, the design of a retrospective study inevitably decreases the level of evidence. The efficacy of the FPS system still needs confirmation from a prospective study. However, our results are sufficient to reach a preliminary conclusion.

In conclusion, wound perfusion in bone and soft-tissue surgery can be assessed by ICGA and graded by the FPS system, which can predict postoperative necrosis and clinical outcomes. Proper measures should be taken based on the FPS system to avoid wound complications.

**Take home message**

- We found wound perfusion in bone and soft-tissue surgery can be assessed and graded by indocyanine green fluorescence angiography.

- We proposed a novel system, known as the Fluorescence Perfusion Scale (FPS), which consists of three types of fluorescence angiography features corresponding to different perfusion statuses and outcomes.

- Intraoperative proposals according to FPS were summarized to guide future application of this technique.

## Data Availability

The data that support the findings for this study are available to other researchers from the corresponding author upon reasonable request.
